# Treatment of evolving cancers will require dynamic decision support

**DOI:** 10.1016/j.annonc.2023.08.008

**Published:** 2023-10

**Authors:** M. A. R. Strobl, J. Gallaher, M. Robertson-Tessi, J. West, A. R. A. Anderson

**Affiliations:** 1 Integrated Mathematical Oncology Department, H. Lee Moffitt Cancer Center & Research Institute, Tampa; 2 Translational Hematology and Oncology Research, Lerner Research Institute, Cleveland Clinic Foundation, Cleveland, USA

**Keywords:** mathematical modeling, treatment scheduling, cancer ecology and evolution

## Abstract

Cancer research has traditionally focused on developing new agents, but an underexplored question is that of the dose and frequency of existing drugs. Based on the *modus operandi* established in the early days of chemotherapies, most drugs are administered according to predetermined schedules that seek to deliver the maximum tolerated dose and are only adjusted for toxicity. However, we believe that the complex, evolving nature of cancer requires a more dynamic and personalized approach. Chronicling the milestones of the field, we show that the impact of schedule choice crucially depends on processes driving treatment response and failure. As such, cancer heterogeneity and evolution dictate that a one-size-fits-all solution is unlikel—dinstead, each patient should be mapped to the strategy that best matches their current disease characteristics and treatment objectives (i.e. their ‘tumorscape’). To achieve this level of personalization, we need mathematical modeling. In this perspective, we propose a five-step ‘Adaptive Dosing Adjusted for Personalized Tumorscapes (ADAPT)’ paradigm to integrate data and understanding across scales and derive dynamic and personalized schedules. We conclude with promising examples of model-guided schedule personalization and a call to action to address key outstanding challenges surrounding data collection, model development, and integration.

## INTRODUCTION

### The state of decision making in the clinic

‘The dose makes the poison’—this principle by Paracelsus, a 16th century physician and pioneer of modern Western medicine, epitomizes the inherent trade-offs of systemic cancer therapy. If given at the right dose, then anticancer agents can overcome disease incurable by surgery or radiotherapy alone; however, given at too high or too low a dose they can incur life-threatening toxicity or be ineffectual. But what is this ‘magic bullet dose’^1^? Current clinical practice is largely guided by the maximum tolerated dose (MTD) paradigm, which emerged from early pioneering work on chemotherapies for the treatment of leukemias.^2–4^ It seeks to maximize the tumor cell kill, and thus the chance of cure, by administering the highest dose which can be given before unacceptable side-effects are observed. This dose is established in phase I clinical trials, and in practice is adjusted only in the case of toxicity, interference with other treatments, insurance questions, or tumor progression.^4–6^

**Figure 1.**
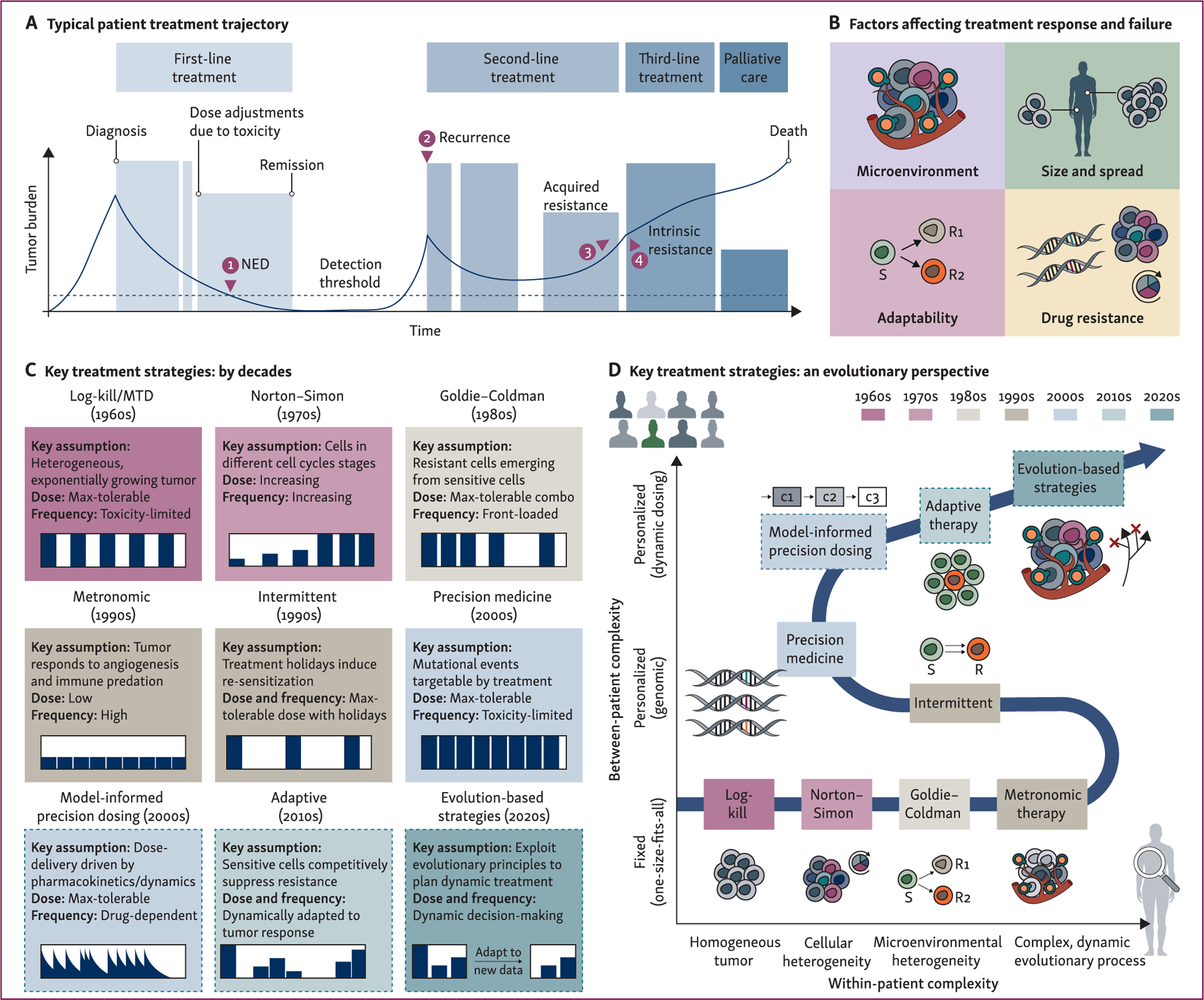
Historical overview of cancer treatment scheduling strategies. (A) Typical timeline for patients under current standard-of-care, maximum tolerated dose (MTD) therapy. During first-line treatment, the tumor is driven to no evidence of disease (NED) (1); the dose is only adjusted for toxicity considerations. However, after some time the tumor recurs (2). Second-line treatment is initially successful at reducing tumor burden, but soon progresses (3), at which point treatment is switched again. Often, recurrent tumors acquire resistance to a therapy or do not respond to subsequent treatments (4). (B) Factors that can affect response to treatment. (C) Summary of key scheduling paradigms, alongside the underlying disease models and examples of the structure of the recommended treatment schedule (black bars represent treatment dosing). Solid bounding boxes denote static schedules in which the schedule is defined at the start of treatment; dashed boxes represent emerging dynamic approaches, currently in early-phase trials, in which the schedule is adjusted according to the patient’s response. (D) History of treatment scheduling, as viewed from our emerging understanding of tumor heterogeneity and evolution. Over time, new approaches have increasingly sought to incorporate a more holistic picture of the tumor and its environment (x-axis). At the same time, other strategies have focused on better tailoring schedules to the individual patient to tackle between-patient differences (y-axis).

Rapid and potentially significant burden reduction is a characteristic feature of a patient with cancer successfully treated at MTD (Figure 1A), and has been pivotal in establishing systemic therapies as a pillar of modern oncology.^3,5^ Yet, two equally defining features are severe toxicity, which results in treatment interruptions and dose modifications, and the fact that all too often improvements are only temporary and/or are only achieved in a subset of the patient’s lesions. For example, >75% of adults treated for acute lymphoblastic leukemia will have no evidence of disease after 4–6 weeks of induction chemotherapy, but only ~30% will be cured.^7^ Upon recurrence this pattern repeats so that most patients will experience a sequence of different agents administered according to MTD principles, but with often progressively shorter lasting success due to the emergence of acquired or intrinsic drug resistance and accumulating toxicity. Eventually treatment may only be given with palliative rather than curative intent, at which point doses may be adjusted to maximize quality of life (Figure 1A).

This illustrates that the MTD paradigm is a crucial tool in oncology, but that on its own it is not enough to address two central challenges in cancer treatment: heterogeneity and evolution. There is mounting evidence that the ‘more is better’ paradigm that motivated MTD for chemotherapies may not apply to targeted and immunotherapies, which can reach their maximum effect before dose-limiting toxicities occur and where adverse events can be harder to quantify.^4,8^ Moreover, cancer is now widely regarded as an evolutionary and ecological disease in which populations of tumor and nontumor cells compete with each other for limited space and resources, are predated upon by the immune system, and can enter symbiotic or parasitic relationships with each other.^9–13^ The MTD paradigm is both evolution-agnostic (administering predetermined fixed dosing schedules) and reactive (altering dose and/or drug only upon a toxicity event or evidence of progression) in nature and often ignores significant contributing factors to disease dynamics such as interactions with the tumor microenvironment, selection for drug resistance, or adaptability (Figure 1B). Considering these factors results in a complex, spatiotemporally varying network of interactions which shapes the relationship between the treatment schedule, outcome, and toxicity in a nonlinear fashion.

The aim of this paper is to argue that the evolutionary nature of cancer necessitates a dynamic and proactive approach to treatment scheduling, informed by mathematical modeling. While the MTD paradigm is effective for short-term tumor burden reduction, more is not always better. Several alternative dosing strategies exist which balance cell killing with other essential objectives, such as favorably re-engineering the microenvironment or preventing the outgrowth of resistance phenotypes. Going forward, we should seek to match each patient with the strategy that will achieve the greatest benefit for their disease, treatment history, and treatment aims. These decisions should also be regularly reassessed over the course of therapy. Furthermore, we aim to bring together key concepts and results from different fields with historically little cross talk, build them into a unifying framework, and promote further integration across disciplines in the quest to tackle the evolutionary nature of cancer. In doing so, we seek to complement ongoing efforts toward personalized medicine (e.g.^14–16^) by proposing to tailor not just *what* drug is used, but also *when and at what dose* it is given.

### Integrative mathematical modeling as a bridge to treatment personalization

All clinical treatment decisions are based on assumptions about underlying biology in the form of a conceptual disease model. For example, even if not necessarily explicitly stated, the MTD paradigm is built on the assumption that the greater the instantaneous cell kill, the greater the likelihood of cure. However, this implicit assumption may neglect important information particular to the cancer, drug, and/or patient at hand.

Personalizing treatment requires a common language to describe concepts across disciplines and integration of quantitative data across time and spatial scales. Mathematics can provide a logical and coherent formalism for clearly identifying our assumptions and mapping them to an outcome.^17^ Specifically, we here refer to so-called mechanistic modeling, which unlike statistical modeling or machine learning seeks not to mimic the data but instead mimic the processes which have given rise to the observed relationships.^18^ For example, while a Cox proportional hazards model gives an equation that links the shape of a survival curve to the treatment that the patient received, this equation contains no information on why and how the treatment altered tumor growth. By contrast, a mechanistic mathematical model of the same data would seek to describe how the treatment affects tumor growth and treatment response over time to give rise to the observed pattern of survival. Mathematical modeling has a long tradition in aiding clinical decision making, with each important advance in treatment scheduling driven by or supported with mathematical models.^19–23^ Mathematical models of cancer are constructed by formulating a set of equations that encode underlying assumptions about the biological dynamics of tumors.^24^ (see e.g. references^19,21,25–31^ for reviews of key models and techniques). In this way, mathematics is necessarily limited by our current biological understanding,^32^ but is a useful construct for identifying testable hypotheses^33^ and deriving quantitative, patient-specific predictions (e.g.^34–40^).

In this manuscript, we envision a future for treatment schedule decision making in which we integrate patient-specific data with eco-evolutionary mathematical models to tackle the complex and evolving nature of a patient’s disease. In the following section, we chronicle key scheduling strategies and show how mathematical modeling has helped to translate conceptual models into concrete dosing plans. We further demonstrate that their differing and sometimes contradictory recommendations for when and at what dose to give treatment can be explained by the fact that each approach is based on a particular tumor model with distinct sets of assumptions which reflect our growing understanding of cancer’s complexity. This will set the stage for the next section in which we use the lens of ecology and evolution to combine these assumptions into a single unified picture of a patient’s ‘tumorscape’. We propose that different historical tumor models can be seen as specific snapshots of this picture representing particular subsets of patients, or the same patient at different points in time. This motivates a more holistic approach of scheduling to address the complex and dynamically changing nature of the disease. In section 4 we present a five-step ‘Adaptive Dosing Adjusted for Personalized Tumorscapes (ADAPT)’ framework with which to better map individual patients to the model and strategy that best matches their current disease characteristics and treatment objectives. To conclude, we describe promising, real-life examples of model-guided schedule personalization and outline key research needs in data collection, model development, and interdisciplinary integration to support quantitative, dynamic decision making in oncology (section 5).

## HISTORICAL OVERVIEW OF ADVANCES IN TREATMENT SCHEDULING

The past decades have not only produced an unprecedented arsenal of anticancer agents, but also a range of ideas as to how these agents should be scheduled (dosing and frequency). In the following, we will extend prior work by Benzekry et al.^22^ and others^21,27,27,30^ and bring together key results from across different disciplines. In doing so, we will show that what at first glance may appear like a ‘model muddle’^41^ is, in fact, a quest connected by a common thread: the aim to better address the complex and dynamically evolving nature of cancer (Figure 1C and D). We propose that as our understanding has grown, new scheduling approaches have been characterized by models with increasing within-patient complexity (advancing left-to-right in Figure 1D), as well as a push for more personalized dosing to better account for between-patient differences (advancing bottom-to-top in Figure 1D). Each of these historical models represents a partial picture of a patient’s disease that we should seek to integrate into a single, evolving whole by using mathematics to systematically translate conceptual models into clinical dosing plans.

### Maximizing cell kill through maximum treatment (Skipper–Schabel–Wilcox, 1960s)

While the first chemotherapy agents were described in the 1940s and 50s, their utility was initially limited because remissions were typically partial and short-lived. In a seminal study, Skipper, Schabel, and Wilcox^2^ showed that in order to achieve more durable responses it was important to carefully consider the treatment schedule. They compared the effect of treating leukemic mice with either a daily, low dose of chemotherapy or a single high dose. They found that the single dose resulted in a greater tumor-burden reduction and a higher probability of cure, even though the low-dose schedule administered a greater cumulative dose. Using mathematical modeling, they^2,42^ and others^43^ subsequently deduced that this was because most leukemia cells in the mice were actively proliferating, making the tumors relatively homogeneous and drug-sensitive, so that treatment at a particular dose always killed the same fraction of cells. This simple math model, known as the ‘log-kill law’, has become a cornerstone of mathematical oncology. Skipper et al.^2,42^ concluded that to maximize the chance of cure, treatment should be given at the highest dose and frequency which toxicity permits (i.e. the MTD; Figure 1C).

These insights motivated Freireich et al^44^ to develop a regimen for pediatric leukemia in which four chemotherapies were combined at maximum dose and at maximum frequency (vincristin, amethopeterin, 6-marptopurine, and prednisone (VAMP)). Their study produced for the first time long-lived remissions, and in some cases cures, in this hitherto untreatable disease.^3,44,45^ This revolutionized the treatment of leukemia and helped to establish MTD chemotherapy and its associated mathematical law as one of the key tools in cancer therapy.^5^

### Overcoming residual disease through treatment escalation (Norton–Simon, 1970s)

Despite the success of the log-kill law in leukemias, translation to solid tumors has been less successful.^3^ Aiming to address these issues, in 1977 Norton and Simon^43^ proposed an updated model to better account for the more complex treatment dynamics and toxicity constraints in these settings (Figure 1C). A number of studies around that time found that the proliferating fraction of cells in solid tumors changed with tumor size.^46–48^ This meant that unlike in Skipper et al.’s work in leukemias,^2,42^ the fraction of cells affected by treatment in solid tumors would change over the course of therapy as the tumor’s growth fraction changed. Using a revised version of the log-kill model, Norton and Simon^43^ showed mathematically that to maximize the chance of cure it was crucial that residual disease was treated at the maximum dose and frequency. For cell-cycle-dependent chemotherapies, this is due to the fact that when cell numbers are small it becomes increasingly unlikely that treatment administration simultaneously overlaps with mitotic phases of the few remaining cells. In addition, they observed that the MTD paradigm results in severe toxicity. In practice, this means that frequently treatment has to be de-escalated over time by either reducing doses and/or delaying the next administration.^5^ Thus, they proposed that in solid tumors, rather than treating at the maximum intensity from the start, treatment should instead be gradually escalated (increasing dose and/or frequency). Moreover, when several drugs are administered, these should be given sequentially to allow maximal intensification of each drug. They hypothesized that in this way it would be possible to treat residual disease at higher intensity, thereby maximizing the chance of cure.^43^

Norton and Simon’s ‘back-loaded’ approach has played an important role in the development of adjuvant chemotherapy protocols.^49,50^ Adjuvant chemotherapy is aimed at eradicating microscopic disease remaining after surgery in order to improve cure rates, in particular in patients in whom the cancer has advanced locally, but has not yet become metastatic. Studies at the National Institutes of Health (NIH) in the 1960s demonstrated that this principle worked in certain patients with breast cancer, yet follow-up trials struggled to further improve results or expand them to a wider population (reviewed in Burotto et al.^50^). Motivated among others by Norton and Simon’s arguments, more intense (higher administration frequency and/or higher doses; also referred to as ‘dose-dense therapy’) and more complex, multidrug regimens were tried.^5,49,50^ For example, Citron et al.^51^ compared adjuvant chemotherapy administered either every 2 or every 3 weeks and found that the more intense, 2-weekly treatment decreased the annual probability of disease recurrence by 26% and the annual probability of death by 31%. Successes such as these have established adjuvant therapy as a key tool of modern oncology and demonstrate how mathematical modeling can help to support the conceptual development of new paradigms. Yet the benefit of adjuvant therapy varies significantly between treatment settings (e.g. the survival benefit of adjuvant chemoradiotherapy in lung cancer is estimated to be ~5% at 5 years^50^), indicating that further research is required on how to choose, schedule, and combine agents to eradicate small cancer populations.

### Tackling heterogeneity by striking early (Goldie–Coldman, 1980s)

While the Norton–Simon model allowed for drug sensitivity to vary with tumor size, it assumed that there was no heterogeneity between tumor cells and that cure was still merely a question of treating long and hard enough. To incorporate emerging research showing that cells could develop intrinsic drug resistance, in the 1980s Goldie and Coldman considered a more complex treatment model to study the impact of a resistant subpopulation on scheduling. Using mathematical modeling, they showed that the probability of a random resistance mutation occurring depends nonlinearly on the tumor size, and increases rapidly the longer the tumor is allowed to expand after diagnosis.^52–54^ They concluded that treatment should be started as early and aggressively as possible to minimize the chance of acquiring resistance (Figure 1C). Moreover, if toxicity does not permit simultaneous administration of multiple drugs, they proved mathematically that the treatments should be rapidly alternated to maximize the chance of a cure.^55,56^ In this way the sizes of different resistant subpopulations could be kept as small as possible, thereby reducing the chance of multidrug resistance.^55,56^ As such, like Norton and Simon, they posited that Skipper et al.’s model was overly simplified, but by focusing on dedicated resistance mechanisms rather than the cell cycle state and by assuming that resistant cells randomly arise from sensitive cells, they drew very different conclusions regarding the best profile of drug dosing over the course of treatment.

However, despite compelling theoretical results, evidence for the clinical utility of the Goldie–Coldman paradigm is limited (see Perry^5^ for an overview). For example, in breast cancer, a number of early studies did indeed report that a delay between surgery and adjuvant therapy is associated with a shorter time to progression (e.g. ^57,58^), consistent with Goldie–Coldman’s recommendation to start treatment as early as possible. However, as these studies have been followed up by larger-scale investigations, evidence for the negative effect of delaying treatment has diminished (e.g. ^59,60^), suggesting that resistance evolution is generally more complex than assumed by the Goldie–Coldman model.

### Looking beyond the tumor (metronomic therapy, 1990s)

While tumor cells are the target of therapy, Folkman, Hannahan, Kerbel and others^61–63^ observed in the late 1990s that tumor growth and treatment response are driven not only by the characteristics of the tumor cells themselves, but also by their environment. Focusing in particular on the emerging role of tumor angiogenesis, they pointed out that administration of drugs at their MTD often requires significant rest periods. These interruptions allow tumor vasculature to recover which helps to drive subsequent recurrence. To avoid such breaks and to better leverage the antiangiogenic effect of chemotherapies, they proposed to administer treatment at a low dose, but at very high frequency, ‘akin to the uninterrupted ticking of a metronome’^64^ (Figure 1C).

Metronomic therapy has since yielded promising results in a range of modeling, preclinical, and clinical studies (see^22,65^ for in-depth reviews). One challenge in developing metronomic schedules is identification of a lowered dose level that is tolerated when given at a high frequency while also remaining effective against the tumor.^66^ Mathematical modeling is ideally suited to address this challenge, as demonstrated by a recent study in which mathematical models were used to derive a metronomic vinorelbine chemotherapy schedule that yielded promising results in a phase I study in lung cancer and mesothelioma.^67,68^ Moreover, further research revealed that metronomic therapy has immunostimulatory effects as lower doses cause less damage to immune cells and induce a more immunogenic form of cell death.^64^ Mathematical models have been used in this context to find the schedule that balances these indirect effects with direct tumor cell kill and toxicity.^69–71^ While MTD approaches remain the mainstay in the clinic, the successes of metronomic therapy have positioned it as a well-established alternative paradigm that demonstrates the benefits of a more holistic view of cancer when designing treatment regimens, particularly for advanced disease.

### Resensitizing the tumor through strategic breaks (intermittent therapy, 1990s)

The indiscriminate nature of cytotoxic (cell-killing) chemotherapies means that these agents can typically be administered for only a few months. As a result, the paradigms discussed so far have been primarily concerned with maximizing response and/or minimizing the chance of relapse following the end of treatment. With the introduction of hormone and targeted therapies, which could be given for longer periods, the focus turned to tackling acquired resistance which caused tumors to regrow despite continued treatment. Preclinical research in different disease settings such as prostate cancer^72,73^ or *BRAF*-mutant melanoma^74,75^ has shown that resistance acquisition can sometimes be slowed and even reversed^75^ if treatment is temporarily withdrawn. This has spurred investigation into intermittent schedules in which treatment is paused at regular intervals with the aim of resensitizing the tumor and improving patients’ quality of life by reducing toxicity (Figure 1C).

However, even with promising preclinical results,^72–75^ clinical testing has so far proven inconclusive.^76–82^ This is also true for so-called bolus-dosing schemes which are a form of intermittent therapy in which treatment is given at doses beyond the MTD to target resistant cells on the basis that the breaks allow these higher doses to be tolerated (e.g.^83–86^). One reason for the challenge in translating preclinical results into practice in this setting could be that different biological mechanisms have been implicated in this resensitization, even within the same cancer type, indicating that it may be driven by a range of factors depending on the particular cancer’s characteristics (e.g. differentiation of cancer stem cells in prostate cancer^72,73^ versus drug addiction^74^ or cell plasticity^75^ in melanoma). As a result, it is plausible that a given intermittent scheme is beneficial only for a specific subset of patients, and others may require a different schedule (e.g. longer or shorter holidays), or will not benefit at all. To address this issue, mathematical modeling has been used to elucidate factors that determine when intermittent therapy is beneficial, and how to potentially tailor treatment based on the longitudinal response dynamics of a patient’s initial cycle (e.g. ^75,87,88^). In this way, targeted therapies have driven treatment personalization not only in terms of which agents are administered (precision medicine) but have also stimulated a discussion about individualization of treatment schedules.

### Personalizing toxicity management (model-informed precision dosing, 2000s)

An even stronger push for mathematical model-based schedule design and potential personalization has been made in the management of drug toxicity. Initially, toxicity was included only as a vague, generic constraint on total drug usage, but over time, these conceptual models have been formalized into elaborate mathematical models of their own (see, for example, the work by Friberg et al.^89^). In addition, the complex processes that regulate drug uptake, distribution, metabolism, and excretion generate a nonlinear relationship between the dosing regimen and the achieved (and achievable) drug level in tumor and nontumor tissue (drug pharmacokinetics). The fields of pharmacometrics and quantitative systems pharmacology use mathematical modeling to quantify and predict exposure levels and toxicity, and have become key components of modern industrial drug development, informing first-in-human doses and dosing regimens.^90–92^ For example, a recent simulation study by Morrissey et al.^93^ showed that 2-weekly and 4-weekly regimens of the immunotherapy atezolizumab were predicted to perform comparably to the approved 3-weekly schedule, which resulted in their subsequent approval by the Food and Drug Administration (FDA) without the need for further large-scale trials.

However, a key challenge is that due to differences in physical (e.g. body weight), genetic/metabolic (e.g. drug transporter polymorphisms), and environmental factors (e.g. diet or concurrent medications) there can be significant pharmacokinetic and pharmacodynamic variability between patients so that a single dose can result in a range of exposures and responses. To account for these issues, more recently so-called *therapeutic drug monitoring* (TDM) and *model-informed precision dosing* (MIPD) approaches have attracted growing attention.^94–96^ By monitoring drug levels along with markers of toxicity (e.g. neutrophil or platelet counts), one can dynamically adjust the treatment dose to ensure both efficacy and safety (Figure 1C). In oncology, TDM or MIPD approaches have mostly been tested in settings with narrow therapeutic windows, where overdosing and under-dosing can have potentially catastrophic consequences, such as methotrexate treatment in children^97–101^ or combination chemotherapy protocols (e.g. ^37,102^; see also section 4.3). As such, while their focus has typically been more on the processes that govern drug pharmacokinetics and its impact on nontumor tissue, these approaches have demonstrated how mathematical models can help to incorporate a more holistic and personalized perspective of the many factors that drive treatment response into schedule design. At the same time, evidence for the large-scale utility and cost-effectiveness of MIPD is still missing, which illustrates some of the scientific, procedural, and regulatory challenges in integrating more dynamic and personalized scheduling into clinical practice.^95,103^

### Leveraging competitive suppression (adaptive therapy, 2010s)

The most recent wave of innovation in scheduling has been inspired by the translation of lessons learned from ecosystem and pest management to cancer, and has continued the trend toward a more complex view of the tumor and the need for dynamic scheduling (Figure 1D). In addition, it has shown that the choice of schedule depends not only on *what* we treat but also on *why* we treat. The most prominent example is that of *adaptive therapy* which aims to establish tumor control rather than cure, particularly in metastatic patients where cure may be unlikely.^104,105^ The central assumption behind adaptive therapy is that drug-resistant cells are in competition with drug-sensitive cells for space and resources, and that potentially they are even at a fitness disadvantage in the absence of treatment due to a cost of resistance (e.g. the energetic costs of running drug efflux pumps). Thus adaptive therapy proposes to dynamically reduce treatment in order to maintain sensitive cells to competitively suppress resistant cells (Figure 1C). The effectiveness of adaptive therapy is dependent on the protocol’s ability to handle the trade-off between negative risks of higher tumor volume and maximizing the competitive suppression of sensitive tumor cell types.^106–108^ Mathematical modeling has been used both to inform this trade-off and to dynamically incorporate new data from patient-specific biomarkers.^88,109^ Indeed, both preclinical experiments^104,110^ and clinical trials^75,111^ of adaptive therapy have relied on mathematical modeling to investigate the role of various assumptions about spatial interactions,^112–115^ cost of resistance,^116^ combination therapy,^117–119^ dose modulation,^120^ and growth dynamics.^121^

In a recent pilot adaptive therapy trial on metastatic castration-resistant prostate cancer (NCT02415621), treatment administration was personalized using a simple rule-of-thumb treatment protocol based on a putative biomarker of tumor burden, prostate-specific antigen (PSA). Treatment stops when the PSA dips below a threshold of 50% of the pretreatment baseline, and is resumed upon reaching or surpassing the baseline. While the study only included 17 patients and was nonrandomized, its findings of a 46% reduction in drug use and a 19.2-month increase in time to progression relative to a historical control cohort are promising.^111,122^ Further clinical trials in, for example, castration-sensitive prostate cancer (NCT03511196), melanoma (NCT03543969), or ovarian cancer are ongoing (NCT05080556).

### Steering tumor evolution (evolutionary therapy, 2020s)

In addition to adaptive therapy, there is a growing list of alternative ‘evolution-based treatment strategies’ which view tumor growth and treatment as a complex, dynamic, evolutionary process requiring proactive personalized dosing (Figure 1C and D). These can be broadly categorized into: (i) extinction therapy, (ii) evolutionary double-bind (evolutionary trap), and (iii) ecological therapies. Extinction therapy draws lessons from mass extinction events from the Anthropocene era^123^ in using a first therapy to massively reduce the size and heterogeneity of a tumor population, followed immediately by a second demographic or environmental strike therapy to render the remaining populations unstable, leading to extinction (cure).^124–126^ Trials have been proposed in Ewing sarcoma^127^ and rhabdomyosarcoma (NCT04388839),^128^ and are actively accruing in prostate cancer (NCT05189457). Another approach, called a double-bind, is an evolutionary strategy where evolved resistance to one drug leads to increased susceptibility to a second drug.^129^ Similarly, collateral sensitivity has emerged as an approach to combat multidrug resistance,^130,131^ and has demonstrated promising results in targeting antibiotic resistance.^132,133^ Evolutionary steering may be possible by exploiting these vulnerabilities,^134,135^ benefiting from the use of mathematical modeling to optimize the timing of switching drugs.^117,119,136–138^

## THE BIG PICTURE: TREATMENT SCHEDULING IS A MULTIFACTORIAL PROBLEM

The previous section illustrates how many different approaches to treatment scheduling have been developed over the past decades. But despite steady gains in scientific knowledge and clinical data collection to inform new assumptions, no single ‘magic bullet’ treatment strategy has emerged. In fact, the recommendations made by different approaches do not generally agree and can even be incompatible (e.g. escalation versus de-escalation over time). We propose that the reason for this discrepancy is the complex, evolving nature of cancer, where many factors acting on different temporal and spatial scales determine how a patient will respond to a given treatment schedule.

### Personalized medicine requires an understanding of the disease characteristics

**Figure 2.**
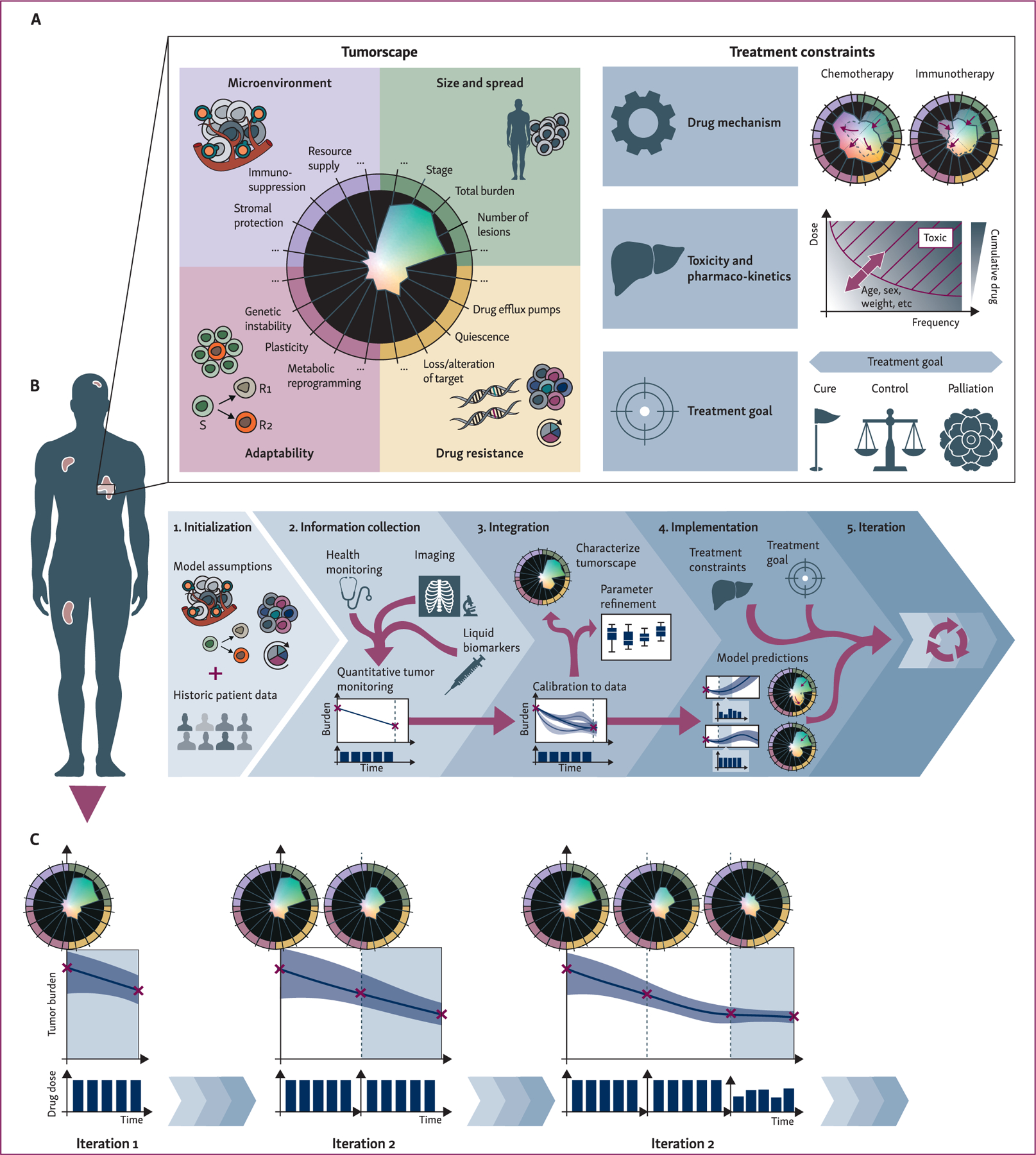
ADAPT: tackling the patient’s evolving tumorscape through model-informed, dynamic scheduling. (A) Choosing the ‘right’ schedule is a multifactorial problem. Broadly, these factors can be classified into two types: (i) features of the ‘tumorscape’, which are the characteristics of the disease that impact the treatment response, and (ii) ‘treatment constraints’ which are other factors that determine whether a particular schedule is feasible, and its outcome is desirable. In this spider plot visualization of the tumorscape, we have exemplified some of the factors that define the tumorscape and arranged them such that growth-promoting characteristics spoke outward. (B) To tackle this complexity, treatment scheduling should be dynamic, personalized, and be supported by mathematical modeling. Such model-informed precision therapy consists of five key ‘ADAPT’ steps. (C) By iterating this process over time, information and decisions are integrated across spatial and time scales into a quantitative model of the patient’s tumorscape, with which treatment decisions can be systematically evaluated to support clinical decision making. ADAPT: Adaptive Dosing Adjusted for Personalized Tumorscapes.

First, we observe that there is a host of factors related to the characteristics of cancer as a disease. These factors can be broadly categorized as relating to (i) the tumor’s size and spread (e.g. tumor volume, number of metastases); (ii) the presence and nature of cell-intrinsic drug resistance mechanisms (e.g. expression of drug pumps, abundance of quiescent cells, or presence of sensitizing or resistance mutations); (iii) the tumor’s ability to adapt and evolve in response to treatment (e.g. the degree of genetic instability, or phenotypic or metabolic plasticity); and (iv) the microenvironment (e.g. the degree of immune suppression or stromal protection). We can visualize this multifaceted nature of a patient’s cancer by using a complex shape in a multidimensional space in which each axis represents one of these traits (Figure 2A): the ‘tumorscape’. The unique oncogenic trajectory and pretreatment history of a patient’s disease means that each patient will be characterized by a slightly different initial shape—their personal tumorscape—which impacts how they will respond to a given treatment schedule (Supplementary Figure S1, available at https://doi.org/10.1016/j.annonc.2023.08.008). For example, a high-dose MTD strategy will work well in a patient with largely sensitive disease but may have only short-lived benefits in patients with pre-existing, cell-intrinsic, or environmental resistance. By contrast, the latter two cases may be expected to have more longer lasting benefits from a metronomic treatment schedule by either selecting for drug-sensitive cells or preserving immune function, respectively (Supplementary Figure S1, available at https://doi.org/10.1016/j.annonc.2023.08.008). In each example, initial differences are subtle but trajectories diverge rapidly, illustrating the importance of continuous monitoring and measurement to characterize cancer.

### Key constraints in treatment scheduling

The second set of factors that have been shown to influence treatment scheduling are related to the treatment itself (Figure 2A, right). First, there is the mechanism of action of the drug, which determines which factors of the tumor and its environment will be impacted. For example, while chemotherapy typically results in immediate and significant tumor size reduction, it will also have negative knock-on effects on nontumor tissue, such as the immune system. By contrast, immunotherapy (e.g. checkpoint inhibitors) may result in less immediate burden reduction, but will promote an immune response that will kill tumor cells over time and can trigger further microenvironmental re-engineering. Second, there are pharmacokinetic and toxicity constraints which determine the temporal characteristics of drug response and impose upper as well as lower limits on which physiological drug levels are achievable. Finally, the recent work on evolutionary therapies has highlighted that subtly, but importantly, we should also carefully think about the reasons for *why* we treat. While cure is the supreme goal of treatment, clinical reality is that many patients cannot be cured with current methods, particularly those with advanced cancers. With the understanding of what is practical and achievable for a given patient, treatment can be tailored to that particular goal. Importantly, that goal may change over the course of treatment.

### Personalized therapy requires dynamic scheduling

Having summarized the many factors that influence how a tumor will respond to a given schedule, we draw two key conclusions for the design of more effective regimens: First, there is no single strategy that works for all patients—not MTD or any of the alternatives we have described. Given the complexity of the tumorscape, focusing just on a subset of factors can be misleading. For example, both the sensitive and the environmental-driven tumor in Supplementary Figure S1, available at https://doi.org/10.1016/j.annonc.2023.08.008, have low levels of cell-intrinsic resistance yet respond differently due to differences in their microenvironments. The multifaceted nature of cancer also explains why the strategies discussed in the previous section, which focused on specific aspects of the disease, have only been effective in a subset of cancers. However, this observation is not a problem, rather it is part of the solution: stratification is key. Truly personalized cancer therapy should not only personalize *what* drug is given, but also *how often and at what dose* it is administered. Second, the complex multiscale nature of cancer and its interaction with therapy means that our treatment approach must be dynamic. As a cancer develops and is subjected to treatment, its other characteristics also change (Supplementary Figure S1, available at https://doi.org/10.1016/j.annonc.2023.08.008). Treatment protocols should respond to changes in the tumor state, and ideally, intentionally steer tumor characteristics to a state favoring elimination or control.

## EMPOWERING CLINICAL DECISION MAKING USING MATHEMATICAL MODELING: THE ADAPT FRAMEWORK

### Complex tumorscapes require quantitative decision making

How do we make an integrative and dynamic approach become reality? Critically, we need tools to better map out the multifaceted nature of a patient’s ‘tumorscape’ and what it implies for treatment. In current clinical practice we build what are essentially conceptual models of the patient’s disease in order to decide treatment. However, as discussed previously, the complex and evolving nature of cancer requires integration of data and processes at multiple temporal and spatial scales, which brings such ‘*in cerebro* modeling’^95^ to its limits. This is similar to the way in which during a game of billiards we have relatively good intuition for what will happen when 2 balls collide, but not for the ball distribution pattern for all 15 balls following a break shot. Further, to keep pace with the evolving nature of cancer, it is essential that we regularly revisit these models and decisions as more information on a patient’s treatment response (or lack thereof) becomes available.

### The ADAPT framework

Here we outline one potential vision for future clinical decision making in which we reinforce current conceptual modeling with mathematical modeling to better integrate our knowledge and use supporting data across scales in a quantitative manner, systematically explore different scheduling options, and update our decisions as more data become available. Mathematical modeling has played an important role in uncovering guiding principles in complex biological systems, ranging from classical ecology to pattern formation on animal coats or protein interaction networks.^139^ Key in this has been an iterative dialog between experiment and model^17,139^ (also known as the ‘systems biology cycle’^140^). We propose to adapt these principles from knowledge discovery to the process of treatment decision making. Figure 2B and C illustrates the decision-making framework, which we will refer to as ADAPT: Adaptive Dosing Adjusted for Personalized Tumorscapes. ADAPT comprises five key steps—the 5 Is of model-informed precision therapy—beginning with Initialization, followed by Information Collection, Integration, Implementation, and subsequent Iteration. To illustrate how ADAPT-based decision making can help to better tackle the complex and evolutionary nature of cancer, we will outline how it could improve the care received by a patient such as the one discussed in Figure 1A.

#### Initialization.

When the patient is first diagnosed, the conceptual model formed by the medical team is translated into a mathematical model (or more likely, a set of plausible models). To do so, the disease biology, the patient’s medical history and diagnostic results, available treatment options, typical resistance mechanisms, and the medical team’s experience are discussed and formalized using a suitable mathematical modeling framework. Depending on the available data and knowledge, such a framework would include, for example, models of the cancer’s molecular evolution, aiming to predict likely resistance mutations,^141,142^ or models of the patient’s cells signaling networks to identify the most effective drug candidate(s) and to assess toxicities,^143^ or models of the interactions between different tumor and nontumor populations, aimed at elucidating long-term eco-evolutionary effects.^122^ In addition, historical data from clinical trials and available medical records are retrieved to provide initial constraints on the model parameters. Together, this information drives a discussion about (i) the initial course of treatment; (ii) the type and frequency of data to be collected during therapy; and (iii) the type and frequency of decision support that will be provided (e.g. detailed dosing plans versus general advice on the order of treatments).

#### Information.

In the next step, the patient receives treatment and is monitored as per the agreed protocol. Longitudinal data are crucial to parameterizing a dynamical system, and as such we anticipate more frequent and dynamic follow-up than is the case in current clinical practice but within practical and financial constraints. In addition, with each cancer and patient, there exist different biomarkers that give insight, including imaging (e.g. computed tomography, magnetic resonance imaging, or histopathology) and blood biomarkers [e.g. protein-based markers such as PSA, cancer antigen 125 (CA125), or cancer antigen 9 (CA9), and emerging liquid biopsy techniques, such as circulating tumor DNA (ctDNA) monitoring]. Health monitoring from clinical data including toxicity and quality of life are also measured and considered. As such, a key part of this step is a continued discussion about, and if necessary revision of, which set of measurements is the most informative, and at what frequency it should be collected to ensure that the measured data and the model are congruent.

#### Integration.

Next, the model is recalibrated using newly collected data (Figure 2B). Importantly, dynamic, mathematical models can integrate data from different sources (e.g. histopathology and blood markers) at different time points, improving the understanding of how observations at different spatial and time scales are connected.^21,33,39,144^ In doing so, mathematical models are also more flexible than statistical models or nomograms in accommodating unforeseen changes in the treatment plan or data collection^145^ (e.g. due to toxicity or timetabling issues). By comparing the fitted model with the data we can assess how well the proposed model explains the patients’ disease, and by comparing model predictions from the previous time point with the new data, we can validate its predictive power.^39^ By using methods such as Bayesian inference,^146–149^ we can thereby piece together in a quantitative and systematic fashion a picture of the patient’s tumorscape, which allows us to better understand the nature of disease we are trying to treat, and how it is changing (e.g. estimating the frequency of treatment-resistant cells in the tumor^150,151^).

#### Implementation.

Once the mathematical model has been calibrated and validated, we can leverage it to systematically interrogate different possible treatment options (Figure 2B). To do so, we first decide what metric, or combination of metrics, we seek to optimize, and over what time frame. Options include ‘standard’ metrics such as the overall tumor burden, toxicity, or financial costs, but also more nuanced considerations such as preserving the sensitivity of the tumor to the next line of treatment. Importantly, the choice of treatment goal can strongly impact the resulting strategy,^152–154^ exemplified by the distinct recommendations made by aggressive MTD therapy, which seeks to eradicate the tumor, and adaptive therapy, which aims to maximize time to treatment failure. In this step we also define and incorporate treatment constraints resulting from drug pharmacokinetics, toxicity, clinical timetabling, among others. In many ways, this process mimics current clinical decision making, except that the use of a mathematical model allows for a more quantitative and systematic evaluation of the different options, using, for example, optimal control.^20,29,155^ There are other mathematical approaches, such as model predictive control, that allow for on-the-fly updating which can be used to continuously provide patient-specific decision support to clinicians taking into account the dynamics of a patient’s disease trajectory (e.g.^100,101,156,157^). These are especially useful when there are many potential treatments, treatment combinations, and dosing strategies to choose from. Furthermore, multiple mathematical tumor models can be considered in parallel to compare treatment courses recommended under different sets of assumptions. Finally, the results on the inferred tumorscape and implications for possible treatment strategies are reported back to the clinical team, for example, in the form of a quantified risk profile^158^ based on data, history, and patient-specific needs. Importantly, we do not propose to replace the clinical team, but rather to support their decision making with additional information from quantitative analysis that considers more of the complexities of cancer that cannot be intuited.

#### Iteration.

After deciding on a certain course of action, we implement it and repeat the circle of information collection, integration, and implementation (Figure 2B). Every treatment decision is accompanied by a plan of when the patient is re-evaluated, which may be a fixed time interval or may depend on the patient’s clinical parameters. Importantly, we re-evaluate and potentially adjust treatment, not only if it performs poorly like for toxicity-triggered dose reductions, but also when it performs well. For example, after an initial treatment that successfully reduces the tumor burden, one might either opt for an aggressive approach to increase the chance of cure or decide to reduce treatment in order to preserve sensitivity. Such iteration is key to keep pace with and eventually outpace the cancer’s evolutionary trajectory.

### Promising real-world examples of model-informed decision making

As we have illustrated in section 2, there is a strong tradition of using mathematical modeling to inform treatment scheduling. In fact, many of the pieces for ADAPT already exist, and there are several notable examples demonstrating that mathematical modeling can be successfully used to directly support clinical decision making. As early as 1972, Sheiner, Rosenberg, and Melmon^159^ suggested that computational pharmacokinetics programs calibrated with population data and refined with data from the individual patient could be used to improve safety and efficacy. Such model-guided ‘therapeutic drug monitoring’^94^ has been explored in a number of settings where there is a high risk of lethal side-effects, such as methotrexate or 6-mercaptopurine treatment in children.^97–101^ For example, in a clinical trial, Evans et al.^97^ found that dosing based on predictions of a simple pharmacokinetics model, that was calibrated with data from the first treatment cycle, was superior to dosing based simply on body weight. Other examples can be found in chemotherapy combination protocols where toxicity is similarly challenging. Meille et al.^87^ recently used a multicompartment mathematical model to optimize the scheduling of the combination of docetaxel and epirubicin in breast cancer, which had resulted in lethal side-effects in a prior study. By fitting the model to data from the first treatment cycle using a Bayesian framework and generating personalized predictions to decide on each patient’s subsequent treatment plan they were able to achieve a more acceptable toxicity profile and promising responses in a phase I/II study.^37,102^

Aside from reducing toxicity, mathematical modeling can help to predict^141,160,161^ and act on cancer evolution in real time.^25,95^ In a recent study,^75^ our group demonstrated that treatment based on a mathematical model of the evolution of sensitive and resistant cells within an individual’s tumor could delay the time to progression in a mouse model of melanoma, which has motivated an ongoing clinical trial (NCT03543969). Most pertinent to how we envision the ADAPT framework to support clinical care is the recently established ‘Evolutionary Tumor Board’ (ETB) at the Moffitt Cancer Center (NCT04343365). The ETB integrates the expertise of mathematicians and evolutionary biologists into the clinical decision support framework of a traditional tumor board, and has enrolled 21 patients at the time of writing.^162^ For each patient in the trial, we collect detailed data about their disease including volumetric imaging, available biomarkers, and other patient history. These data are used in ‘virtual clinical trials’, where the real patient is compared with virtual patients that have been constructed based on historical cohort data from the same disease.^163^ Each virtual patient is a unique parameterization and realization of a disease-specific mathematical model that has been developed for the ETB. Once an analogous virtual cohort is selected, treatment options are simulated within the cohort to provide bounds on the expected response to different therapy options available to the oncologist. When an evolutionary strategy is implemented, a key aspect of the ETB is that it is iterative: we update the model predictions each time we receive new data from the patient. These new data constrain the cohort of matching virtual patients, and may alter the predicted trajectories and therefore alter the decision support information provided by the framework.

## THE EVOLUTIONARY PATH TO TRANSLATION

The ADAPT framework is designed with evolutionary and ecological processes in mind. Some features of ADAPT have already been implemented in the clinic, and calls for using mathematical modeling to support clinical decision making have been made for many years and across various disciplines (e.g.^23,27,29,30,103,143,159,164–167^). While this illustrates that what we envision is possible, none of the examples we have discussed have been adopted in clinical practice, making it clear that this journey has only just begun. We believe that our growing understanding of the heterogeneous and evolving nature of cancer necessitates more quantitative decision making, even if imperfect. Thus we outline key challenges that need to be addressed to fully realize the potential of this framework.

### Mapping out the tumorscape with more of the right data

One of the biggest challenges to implementing these methods into clinical practice is the lack of quantitative temporal data that are necessary to produce a reliable, validated, and predictive model. Standard practice often front-loads data collection (biopsies, imaging), with many disease characteristics of the tumorscape being measured rarely or not at all. Treating a multifactorial disease with evolutionary underpinnings will require increased effort in collecting multiscale, longitudinal data for patients. Taking measurements at different time points along the patient’s trajectory allows for better estimation of parameters for disease-specific dynamics, and measuring across different spatial scales allows incorporation of both the large-scale tumor dynamics (e.g. radiographic tumor burden) and smaller-scale tumor components (e.g. cell-scale phenotypic heterogeneity).

However, more data only translates into more understanding if it is the right kind of data. Merely increasing the resolution along one axis of our picture of the tumorscape may do little for our ability to predict how this tumor will evolve in response to any given treatment. Instead, we need to better understand the eco-evolutionary processes driving the tumor’s trajectory: the *why* rather than just the *what* (e.g.^168–170^). To do so, it is important to increase our ability to follow the tumor’s evolution over time, and to understand the ecological context within which the cells grow, using methods such as tumor phylogenetics,^11,171^ liquid biopsy,^172–174^ spatial transcriptomics,^175,176^ and histoecology.^177^ We acknowledge that many of the factors outlined in Figure 2A are not directly measurable with current technologies, but we hope that by providing a conceptual framework we can guide future discussion about how and when to collect data to best support treatment scheduling decisions. At the same time, an open dialog between the clinicians and the modelers will be necessary to optimize the patient financial and time costs of collecting ever more data.

Hand-in-hand with collection, we need to think about infrastructure to store, manage, and make accessible the data we collect. This applies to both when seeking to apply ADAPT and in the run-up when we require data to develop, calibrate, and validate models and tools. Initiatives such as Project DataSphere,^178^ the YODA Project^179^ or Vivli^180^ illustrate how making data from clinical trials accessible to the public can help to develop new predictive mathematical models. Wider availability of longitudinal historical data will be key for enabling more quantitative decision making in the future.

### Dropping the bullet: learning to tackle cancer’s complexity

The concept of a ‘magic bullet’ has fascinated cancer research for many decades.^1^ However, with few exceptions, a single, simple solution to cancer remains elusive. The same holds true for treatment scheduling: none of the many treatment paradigms we reviewed here are right or wrong *per se,* but each represents a tool for a particular set of circumstances depending on the patient’s tumorscape. What we therefore require is a paradigm shift away from searching for a single approach to cure all patients and the development of tools to compare and integrate different plausible models for an individual patient and identify the strategy that is best for them at a given moment in time.

Variation exists across both spatial and temporal scales. On the population level, patients are characterized by distinct underlying tumor biologies. This heterogeneity has started to be addressed by the use of so-called virtual^181,182^ or ‘phase I’^183^ clinical trials in which each patient is assumed to be represented by a unique parameterization of the same mathematical model. Such virtual cohorts can be used to assess the effect of interpatient variability on the outcome of different treatment protocols, and have been used to optimize oncolytic virotherapy,^184^ stratify patients with melanoma,^163^ and individualize treatments in breast cancer.^185,186^ However, variability also exists within the individual patient where different mechanisms may be at play within different regions of the tumor or at different time points during the treatment trajectory. One solution that we might borrow from weather prediction is the use of ‘spaghetti models’ to evaluate uncertainty in forecast predictions based on an ensemble of models.^187^ However, while a few examples of such multimodel approaches exist,^188–190^ they are rare, and many open questions remain as to how this technique would be integrated within ADAPT, such as the following: (i) How do we choose the candidate models so that they are the most informative? (ii) What criteria do we use to compare the models? and (iii) How do we integrate possibly conflicting recommendations from different models? In addition, while a decision to, or not to, evacuate does not alter the path of a hurricane, our treatment choices do change the tumor’s trajectory, generating complex feedback dynamics. A key first step in addressing these questions will be to generate a more systematic understanding of how existing models for specific disease sites or treatment settings compare qualitatively and quantitatively, in particular with regard to implications for scheduling (e.g. ^26,191,192^).

Another important question which arises in this setting is how to decide treatment in light of multiple metastatic lesions with possibly differing biologies? We believe that again systematic characterization, modeling, and prediction using ADAPT principles can help to better understand which lesions represent the greatest risk, and how treatment should be chosen so as to achieve the most benefit. However, to be able to do so it will be necessary to (i) better characterize metastatic disease in the clinic^193,194^; (ii) develop mathematical models of the interplay between lesions (building for example on^195–197^); and (iii) explore how different treatment strategies perform in light of multiple, heterogeneous lesions.^109,198,199^ Similarly, for simplicity we have represented treatment as a single therapy in this paper, but in practice combinations or sequences of drugs are often given. An important and open question in multidrug therapy is how different lesions, and regions within the same lesion, might differentially respond to therapy. We need to develop tools to better understand and predict these complex responses, which invites the integration of molecular omics measurements of tumor ecology and evolution with mechanistic, mathematical models (e.g. ^200,201^).

### Darwin’s butterflies: identifying the limits on predicting tumor evolution

While an eco-evolutionary view of cancer reveals unifying patterns between the disease in different patients, the unique molecular profile of each tumor raises a crucial question: how predictable is a particular patient’s trajectory^141,202,203^? Mutational events are stochastic. In addition, in complex, interconnected systems small uncertainties in initial conditions or parameters can become vastly amplified over time (also known as the ‘butterfly effect’). This phenomenon is intimately familiar to us from weather forecasting and necessitates that we choose carefully what and how far into the future we seek to predict. For example, when only sparse system-level data are available we may need to limit predictions to whether or not a patient is likely to progress over the next cycle of therapy,^88,109^ whereas when we have detailed imaging it may be possible to derive maps of which regions within a tumor are more likely to respond than others.^204,205^

To inform treatment response, we need to better understand limitations to characterize a tumor’s composition and evolvability. This requires the development of more experimental techniques such as barcoding,^206^ HYPERflasks,^134^ or confetti mice^207^ with which we can test our ability to forecast and manipulate tumor evolution in experimental evolution studies (e.g.^208,209^). In addition, on the modeling side, we need to develop procedures to assert the robustness of proposed treatment protocols (see ^182,210,211^ for examples) and devise contingency plans for when things do not go as planned. As even a short forewarning can help to save lives ahead of an approaching hurricane, also an imperfect disease forecast can be useful—as long it allows us to draw the right conclusions.

### Building bridges: collaboration is required

The examples of model-guided treatment designs discussed in this paper are promising and in our opinion lay the groundwork for the future. But they are, and have typically remained, isolated case studies. Large-scale trials showing the benefit of MIPD, adaptive, or evolutionary therapy over current static approaches are as of yet missing. This is because of the financial, logistic, and regulatory challenges involved in scaling up these approaches to large numbers of patients, possibly treated at different institutions and by different care teams (see also^95,103,212^). For dynamic decision making we require regular monitoring, but many such assays are expensive (e.g. ctDNA or magnetic resonance imaging scans) and only covered by insurance at the few time points foreseen in current clinical care, and may not yet have the approval required for use in decision making. Furthermore, translating incoming measurements into model predictions and subsequent updates to a patient’s schedule in real-time is a complex, resource-and training-intensive process that requires nonstandard resources (e.g. mathematical modeling support) and close-knit, interdisciplinary communication. At the same time, given the focus is on optimizing the use of already approved drugs, in potentially narrowly defined subpopulations, financial support from drug companies can be challenging to obtain.

How can we overcome these difficulties? We believe the answer is collaboration. First, there is the dialog between theoreticians and clinicians. As put by Harrold and Parker^213^: ‘a clinically relevant but mathematically suboptimal solution is superior to a mathematically optimal solution that is ignorant of clinical considerations’. Maintaining feedback between the clinic and the theoretical work will keep the models relevant to existing and novel aspects of the treatment selection process. Simultaneously, such feedback is crucial for building the interdisciplinary infrastructure and acquiring the institutional, financial, and regulatory support to permit larger-scale trials. It can also help to identify new areas for collaboration and thereby widen the opportunities for clinical testing. For example, ‘window-of-opportunity trials’ (e.g. ^214^) or emerging ctDNA-based interventional studies (e.g. ^215^) demonstrate the benefit of adjusting treatment based on the tumor’s response. There could be natural synergy in complementing these efforts using the language of evolutionary therapy and the predictive capabilities of mathematical models. At our institution, this approach has led to mathematics-driven clinical trials, an ETB,^162^ and 10 years of collaborative workshops.^216^ Clinical trials are either planned or ongoing in rhabdomyosarcoma (NCT04388839; phase II),^128^ castration-sensitive prostate cancer (NCT03511196; early phase I), castration-resistant prostate cancer (ANZadapt; NCT05393791; phase II), advanced BRAF mutant melanoma (NCT03543969; early phase I), ovarian cancer (ACTOv trial; NCT05080556; phase II), and advanced basal cell carcinoma (NCT05651828; early phase I). For a review on the role of mathematical modeling within these trials, see West et al.^217^

Second, we advocate for more collaboration between researchers working on treatment scheduling. We have illustrated that a wide range of disciplines, ranging from clinical pharmacology to mathematics and evolutionary biology, has contributed to our understanding, yet historically cross talk between these communities has been limited. One main aim of this perspective was to change this by bringing together key contributions from across fields and by suggesting a common framework to stimulate conversation. We believe that the key to success will be to integrate our experiences over an ensemble of cancers, drugs, models, and approaches (e.g. molecular evolution models,^142^ protein network models,^143^ or mechanical models^40^). Only in this way can we fully address the multiscale and evolving nature of cancer.

## CONCLUSION

While the dose makes the poison, what that dose is, and what it does, depends on a plethora of factors—and changes over time. Recently, the FDA released a call-to-action to revisit the decade-old dose selection process to better address the complex and evolving nature of cancers.^218^ This call illustrates the urgent need to develop methods that account for evolutionary and ecological processes when designing dose schedules for systemic cancer treatment.^218–220^ However, choosing the ‘right’ schedule is a complex, multifactorial problem that requires integration of information across multiple spatial and temporal scales. Research to date has focused on different subsets of these factors and has developed multiple promising (sub)solutions. Next, we need to integrate these. Our perspective is that this can only be achieved through the iterative integration of mathematical modeling into treatment decision support to leverage evolutionary insight and transform treatment scheduling into a dynamic and patient-specific paradigm.

## Supplementary Material

1
