# Supplementary figures and images for: Treatment of evolving cancers will require dynamic decision support
Source: Ann Oncol. Author manuscript; Available in PMC 2023 Nov 30. (PMC10688269; doi:10.1016/j.annonc.2023.08.008)

## MTD Chemotherapy

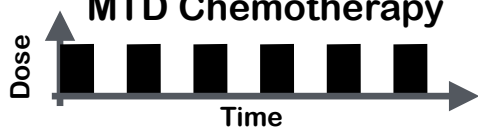

Mostly Sensitive

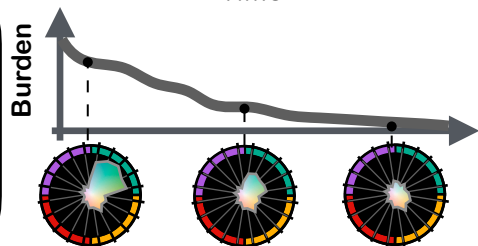

Pre-existing Resistance

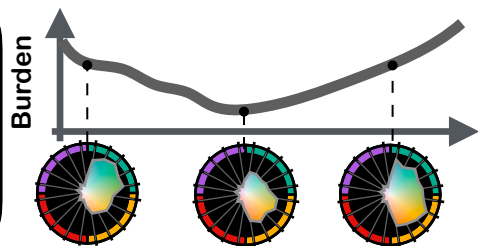

Stroma-driven

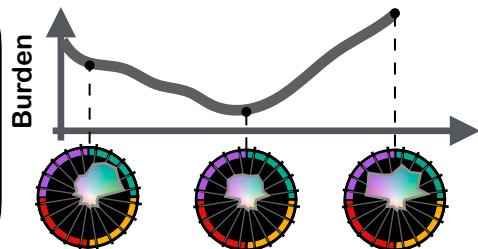

## Metronomic

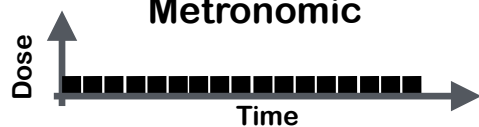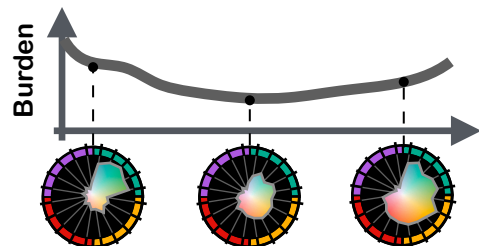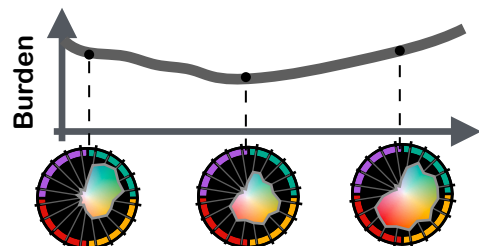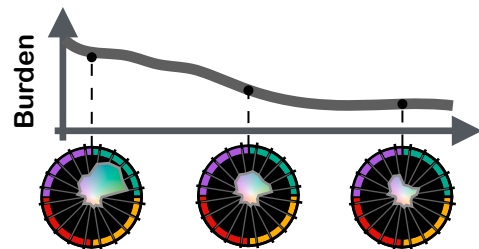

Supplement: 1 [file NIHMS1935438-supplement-1.pdf]
